# The effectiveness of extracorporeal membrane oxygenation in a patient with post myocardial infarct ventricular septal defect

**DOI:** 10.1186/s13019-016-0537-5

**Published:** 2016-09-26

**Authors:** Jintae Kwon, Donghyup Lee

**Affiliations:** 1 Department of Thoracic and Cardiovascular Surgery, Bundang Jesaeng Hospital, Seongnam-si, South Korea; 2 Department of Thoracic and Cardiovascular Surgery, Yeungnam University Hospital, 317-1 Daemyung 5 dong, Namgu, Daegu South Korea

**Keywords:** Post infarction ventricular septal defect, Extracorporeal membrane oxygenation, Cardiogenic shock

## Abstract

**Background:**

Post infarction ventricular septal defect (VSD) is an uncommon but life threatening complication of acute myocardial infarction.

**Case presentation:**

A 62-year-old woman was admitted with acute myocardial infarction (AMI). However, the day after angioplasty and stenting, Transthoracic echocardiography (TTE) showed post infarction VSD. We decided to insert an extracorporeal membrane oxygenation (ECMO) device for stabilization purposes before surgical repair. After 4 days from the implantation, we performed surgical repair successfully.

**Conclusions:**

When optimal medical treatment fails to stabilize a patient in cardiogenic shock, peripheral ECMO could be used as a bridge to definitive surgical therapy.

## Background

Rupture of the ventricular septum after acute myocardial infarction (AMI) is a serious complication. In the pre-thrombolytic era, its incidence was 1–2 % but the use of thrombolytic agents reduced it to 0.2 % [1, 2]. The mortality rate with only medical treatment reaches 90–95 %, but the rate varies from 19 to 60 % with surgical intervention [1].

Despite optimal medical treatment, when ventricular septal defect (VSD) related cardiogenic shock causes rapid deterioration, surgical repair is the only definitive treatment. However, early surgical repair is difficult and complicated due to the presence of friable, infarcted, necrotic tissue.

To defer surgery in patients with cardiogenic shock, peripheral ECMO could be used as a temporary bridge to definitive surgical repair after post AMI VSD.

## Case presentation

A 62-year-old woman with a history of hypertension was admitted with complaints of chest pain and shortness of breath of 3 days duration. Her heart rate was 89 bpm, blood pressure 105/70 mmHg, and temperature 36.5 °C. Chest X-ray revealed cardiomegaly and pulmonary edema. On admission, blood tests revealed Hb 10.9 g/dl, troponin I 5.26, CK-MB 4 ng/ml. Arterial blood gas revealed pH 7.32, pCO 2 21.6, pO 2 80.2 mmHg, bicarbonate 11 mmol/l and SaO 2 92.8 % on room air. Electrocardiography (ECG) showed ST-segment elevation and Q waves in leads V1-4 and coronary catheterization revealed total proximal occlusion of the anterior interventricular branch of the left coronary artery. Angioplasty and stent implantation were performed, and chest pain, shortness of breath, and general malaise disappeared after the procedure. However, the day after angioplasty and stenting, the patient suddenly experienced fatigue, dyspnea, and tachycardia. At this time, her blood pressure was 70/50 mmHg and heart rate 120 bpm. On physical examination, auscultation revealed a 3/6 pan-systolic murmur in the left parasternal area.

**Fig. 1:**
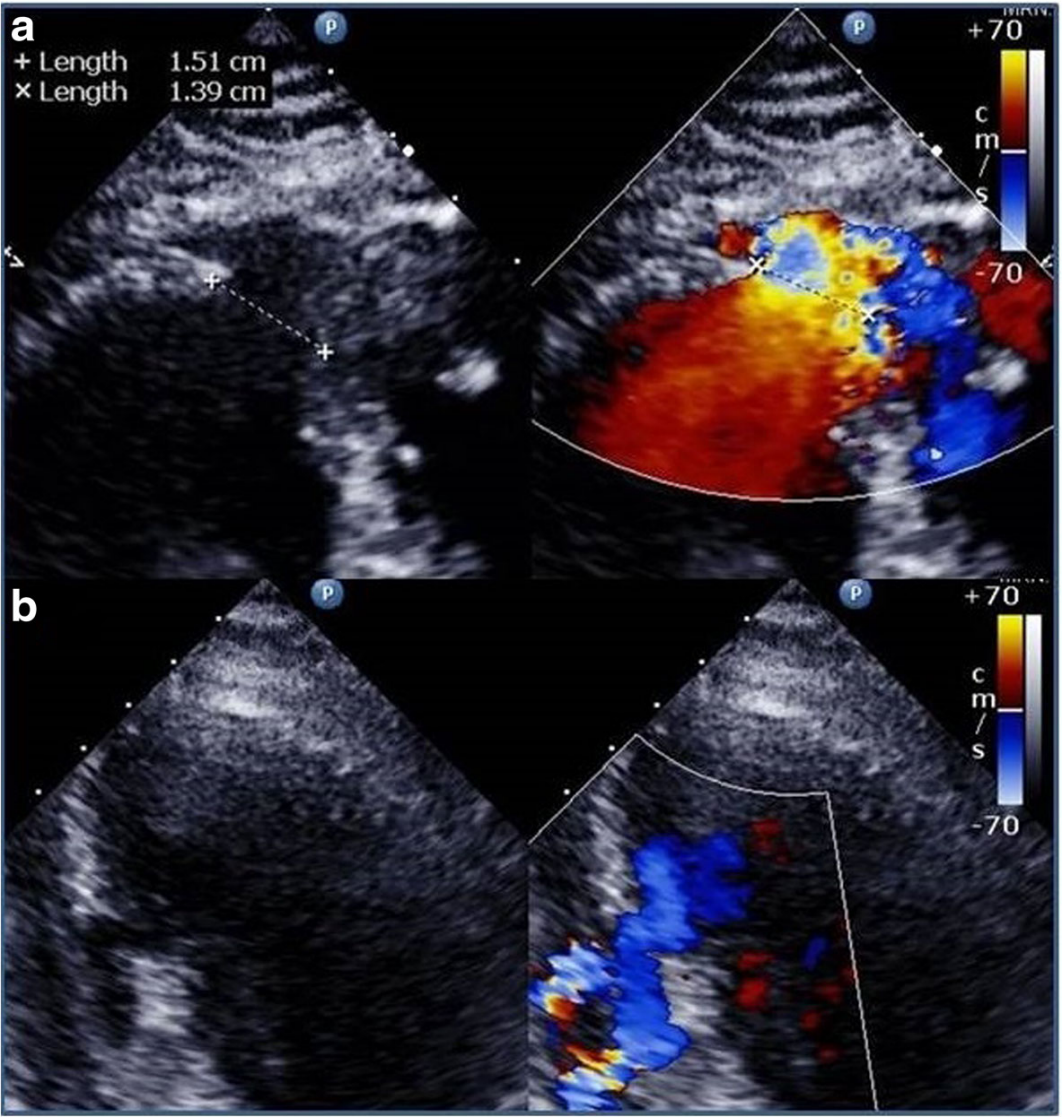
Preoperative echocardiographic findings. **a** TTE in four chamber view showing a 15 mm VSD in the apicoanterior ventricular septum with left to right shunt. **b** Color flow Doppler image showing the 8 mm VSD in the mid anterior ventricular septum

Transthoracic echocardiography (TTE) with Doppler showed an akinetic area in the apex, an ejection fraction of 43 %, and confirmed 15 mm and 8 mm VSDs in the apicoanterior and mid anterior ventricular septum (Fig. 1). Tricuspid regurgitation was mild with moderate pulmonary arterial hypertension with systolic pulmonary artery pressure of 50 mmHg. The total pulmonary to total systemic blood flow ratio (Qp/Qs) of VSD was 5.7.

Symptoms of congestive heart failure progressed due to low cardiac output, and the patient was transferred to the intensive care unit to treat the heart failure and to prevent progression of cardiogenic shock. After her intubation, we administered maximum dose of inotropics and vasopressors. Despite optimal medical treatment, pulmonary hypertension and pulmonary congestion gradually increased, and urine output decreased. We decided to insert an extracorporeal membrane oxygenation (ECMO) device for stabilization purposes before surgical repair, and ECMO support was emergently initiated via a left femoral artery and right femoral vein. Arterial and venous cannulations were performed percutaneously, and distal limb perfusion was achieved with an introducer sheath (7 Fr). Stabilization of the patient was immediate and her clinical status improved. Accordingly, surgical VSDs repair was postponed. After 4 days from the implantation, signs of left leg ischemia developed; hence, we decided to perform surgical repair. On the same day, closure of the VSDs was performed using two patches of bovine pericardium by bilateral ventriculotomy. Using interrupted mattress suture, one patch was placed on the left ventricular (LV) side and the other on the RV side of the VSD. Lastly, both ventriculotomies were closed using Teflon strips to buttress the suture lines. Cardiopulmonary Bypass (CPB) and aortic cross clamp (ACC) times were 219 and 194 min, respectively. Immediately following the surgery, the patient was stable and had a good urine output. Her postoperative course was uneventful. On the third postoperative day, the patient was extubated and transferred to the general ward on the fourth postoperative day.

The TTE prior to discharge showed a residual 6 mm VSD in the mid anterior ventricular septum, and at this time, the total pulmonary to total systemic blood flow ratio (Qp/Qs) of the residual VSD was 1.1. However, the patient’s hemodynamics were stable. The patient was discharged on the fourteenth postoperative day without symptoms of heart failure.

After thirty months postoperatively, the patient remained in good condition clinically. The patient’s blood pressure was well controlled and renal function was normal. Follow-up TTE revealed no significant changes.

## Discussion

Post AMI VSD is a serious clinical problem with a high mortality rate; thus, the management of patients with post AMI VSD is challenging. IABP (intra-aortic balloon pump) provides afterload reduction for the left ventricle, increase in diastolic perfusion of coronary arteries, and decrease in left to right shunting. However, in patients presenting with cardiogenic shock following AMI, the use of a percutaneously placed LVAD (left ventricular assist device) is more feasible, safe, and provides superior hemodynamic support than the standard treatment using IABP [3]. In our hospital, for the patients with post-MI VSD with refractory cardiogenic shock, peripheral VA ECMO is the quickest, the easiest, and the least invasive ventricular support device.

When medical treatment is applied alone, mortality reaches 90–95 %, whereas for surgical repair mortality varies from 19 to 60 % [1]. Thus, surgical repair of post infarction VSD is the definitive treatment of choice [4]. Percutaneous closure using a septal occluder device can be the primary treatment for anatomically suitable patients. Unfortunately, we did not have any experiences with percutaneous interventions nor closure devices.

A recent publication released by the Society of Thoracic Surgeons National Database showed that patient mortality is significantly dependent on the timing of surgery. Patients that underwent surgery within 7 days of symptoms onset were found to have a 54 % mortality rate, but when surgical repair was delayed for more than 7 days after VSD post AMI, the mortality rate fell to 18 % [5]. However, these rates may be unduly biased, because early surgery is usually performed in the patients with marked hemodynamic instability and circulatory compromise.

After AMI, time is needed for the regeneration of heart muscle tissues in the necrotic area [6].

During the early phase of post AMI, infarcted myocardium is weak and friable, and this may prevent appropriate stitching of patch materials around a septal rupture. Consequently, stitches may be pulled out of septal tissue, which increases the risk of tearing and recurrent septal defects. Accordingly, the more chronic VSD is, the easier it is to repair because the septum is well scarred and a patch can be securely sutured.

However, it should be borne in mind that when patients with VSD related cardiogenic shock deteriorate rapidly, surgical repair is the only definitive treatment. We would recommend implementing resuscitative measures initially, especially in patients with cardiogenic shock.

## Conclusions

In conclusion, veno-arterial (VA) ECMO may be an effective means of providing temporary assistance in patients with post AMI VSD related to cardiogenic shock. We suggest when optimal medical treatment fails to stabilize a patient in cardiogenic shock, peripheral VA ECMO could be used as a temporary bridge to definitive surgical repair.

Further studies are needed to assess the effectiveness of ECMO in this patient population.

## Abbreviations

ECMO: Extracorporeal membrane oxygenation
TTE: Transthoracic echocardiography
VSD: Ventricular septal defect
